# Matrix Metalloproteinase-3 Causes Dopaminergic Neuronal Death through Nox1-Regenerated Oxidative Stress

**DOI:** 10.1371/journal.pone.0115954

**Published:** 2014-12-23

**Authors:** Dong-Hee Choi, Ji-Hye Kim, Joo-Ha Seo, Jongmin Lee, Wahn Soo Choi, Yoon-Seong Kim

**Affiliations:** 1 Center for Neuroscience Research, Institute of Biomedical Science and technology, Konkuk University, Seoul, 143-701, Korea; 2 Department of Medical Science, Konkuk University School of Medicine, Seoul, 143-701, Korea; 3 Department of Rehabilitation Medicine, Konkuk University School of Medicine, Seoul, 143-701, Korea; 4 Department of Immunology and Physiology, Functional Genomics Institute, College of Medicine, Konkuk University, Chungju, 380-701, Korea; 5 Burnett School of Biomedical Sciences, College of Medicine, University of Central Florida, Orlando, Florida, 32827, United States of America; University of Pecs Medical School, Hungary

## Abstract

In the present study we investigated the interplay between matrix metalloproteinase 3 (MMP3) and NADPH oxidase 1 (Nox1) in the process of dopamine (DA) neuronal death. We found that MMP3 activation causes the induction of Nox1 via mitochondrial reactive oxygen species (ROS) production and subsequently Rac1 activation, eventually leading to Nox1-derived superoxide generation in a rat DA neuronal N27 cells exposed to 6-OHDA. While a MMP3 inhibitor, NNGH, largely attenuated mitochondrial ROS and subsequent Nox1 induction, both apocynin, a putative Nox inhibitor and GKT137831, a Nox1 selective inhibitor failed to reduce 6-OHDA-induced mitochondrial ROS. However, both inhibitors for MMP3 and Nox1 similarly attenuated 6-OHDA-induced N27 cell death. RNAi-mediated selective inhibition of MMP3 or Nox1 showed that knockdown of either MMP3 or Nox1 significantly reduced 6-OHDA-induced ROS generation in N27 cells. While 6-OHDA-induced Nox1 was abolished by MMP3 knockdown, Nox1 knockdown did not alter MMP3 expression. Direct overexpression of autoactivated MMP3 (actMMP3) in N27 cells or in rat substantia nigra (SN) increased expression of Nox1. Selective knockdown of Nox1 in the SN achieved by adeno-associated virus-mediated overexpression of Nox1-specific shRNA largely attenuated the actMMP3-mediated dopaminergic neuronal loss. Furthermore, Nox1 expression was significantly attenuated in *Mmp3* null mice treated with N-methyl-4-phenyl-1,2,3,6-tetrahydropyridine (MPTP). Together we established novel molecular mechanisms underlying oxidative stress-mediated dopaminergic neuronal death in which MMP3 activation is a key upstream event that leads to mitochondrial ROS, Nox1 induction and eventual dopaminergic neuronal death. Our findings may lead to the development of novel therapeutic approach.

## Introduction

In Parkinson's disease (PD), the dopamine (DA) neurons in the substantia nigra (SN) undergo degeneration. These DA neurons are particularly vulnerable due to the presence of ROS-generating molecules, including DA itself and iron, as well as low antioxidants. Increasing lines of evidence have linked matrix metalloproteinase (MMP) to the pathogenesis of neurodegenerative diseases such as Alzheimer's and Parkinson's disease [1]–[4]. Our previous studies demonstrated that MMP3 plays a crucial role in degeneration of DA neurons in the SN [4], [5]. The active MMP3 is accumulated in the cytoplasm of DA cells under various stress conditions, which was responsible for DJ-1 degradation and abolished its antioxidant property [6] as well as increased alpha-synuclein toxicity by generating C-terminal fragments [7].

A wide range of oxidative damage to cellular macromolecules in nigrostriatal DA neurons, including lipids [8], proteins [9], and nucleotides [10], has been observed in postmortem brains of PD patients. Increasing evidence has suggested that the family of Nox, the enzyme complex that transports electrons across the plasma membrane and generates superoxide, plays a major role in generating ROS in cells [11]. Previously we have shown that the induction of Nox1, an isoform of the Nox family, and ROS generation in dopaminergic cells under various stress conditions such as paraquat or 6-OHDA treatments are crucial for dopaminergic neuronal cell death both *in vivo* and in cell cultures [12], [13].

Previous studies have reported that mitochondria, which have long been considered as a major source of ROS, play a key role in Nox1-mediated superoxide generation [14]–[16]. Mitochondrial ROS are essential but not enough to promote cell death, which requires the sustained accumulation of ROS by the subsequent action of Nox1 [15]. Interestingly, Radisky *et al*. have demonstrated that MMP3 can lead to genomic instability and induce epithelial-mesenchymal transition (EMT) in mammary epithelial cells through mitochondrial ROS generation [17]. They also showed that MMP3 can stimulate EMT of mouse mammary epithelial cells through increased expression of Rac1b, a protein that forms a complex with Nox and promotes the production of ROS [17]–[19].

In the current study, we have investigated the effect of MMP3-derived mitochondrial ROS on the expression and activation of Nox1 in DA cell degeneration. We demonstrated that 6-OHDA-treated N27 cells and MPTP-treated mice show noticeable increase in Nox1 expression via MMP3-mediated production of mitochondrial ROS, and that Nox1 levels and DA neuronal loss are reduced in MMP3 knockout mice. Overexpression of active MMP3 in DA neurons in the SN induced DA cell degeneration and it was significantly reversed by Nox1 knockdown.

## Materials and Methods

### Materials

Fetal bovine serum (FBS), horse serum, RPMI 1640, L-glutamine, trypsin/EDTA, and penicillin–streptomycin, neurobasal medium, and B-27 were from GibcoBRL (Gaithersburg, MD, USA). Protease inhibitor cocktail, Brij35, 3-(4,5-Dimethylthiazol-2-yl)-2,5-diphenyltetrazolium bromide (MTT), dihydroethidine, apocynin, NADH, NADPH, and lucigenin were purchased from Sigma Chemicals (St. Louis, MO, USA). PhosSTOP phosphatase inhibitor cocktail tablets and Avian Myeloblastosis Virus (AMV) reverse transcriptase were purchased from Roche Life Science (Roche Applied Science, Indianapolis, IN). GKT137831 was purchased from Aurora Fine Chemical (San Diego, CA, USA). N-isobutyl-N-(4-methoxyphenylsulfonyl) glycyl hydroxamic acid (NNGH) and MMP3 fluorescent assay kit were purchased from Biomol (Plymouth Meeting, PA, USA). MitoSox Red, 2',7'-dichlorodihydrofluorescein diacetate (DCFDA), tetramethyl rhodamine methyl ester (TMRM) and Amplex Red hydrogen peroxide kit, Trizol reagent, Lipofectamin 2000 were purchased from Invitrogen (Carlsbad, CA, USA). Mouse monoclonal anti-tyrosine hydroxylase (TH) antibody (MAB318) was obtained from EMD Millipore (Billerica, MA, USA) and rabbit anti-malondialdehyde (MDA, ab6463) antibody were obtained from Abcam (Cambridge, MA, USA), goat polyclonal anti-MMP3 (C-19) and rabbit polyclonal anti-Nox1 (H-75) antibody were from Santa Cruz Biotechnology (Santa Cruz, CA, USA). Anti-goat IgG and anti-rabbit IgG were from Sigma Chemicals and Alexa Fluor 488 donkey anti-mouse IgG, Alexa Fluor 488 donkey anti-rabbit IgG, Alexa Fluor 546 donkey anti-mouse IgG, Alexa Fluor 546 donkey anti-rabbit IgG, and Alexa Fluor 647 donkey anti-rabbit IgG were from Invitrogen (Grand Island, NY, USA). Recombinant MMP3 containing only the catalytically active domain (22 kDa) was purchased from Calbiochem (San Diego, CA, USA). Enhanced chemiluminescence kit was obtained from Pierce (Rockford, IL, USA). All other chemicals were of reagent grade from Sigma Chemicals or Merck (Rahway, NJ, USA).

### N27 cell culture

N27 cells are derived from rat mesencephalon and express tyrosine hydroxylase and dopamine transporter [20]. Cells were grown in RPMI 1640 containing 10% FBS, 100 IU/l penicillin, and 10 µg/ml streptomycin at 37°C with 5% CO_2_ supply in humidified atmosphere. For experiments, the cells were plated on polystyrene tissue culture dishes at a density of 2×10^4^ cells/well in 96-well culture plates, 1×10^5^ cells/well in 24-well culture plates, 1×10^6^ cells/well in 6-well culture plates, or 2×10^6^ cells/100 mm plate. After 24 h, the cells were fed with fresh medium and treated with 50 µM or 100 µM of 6-OHDA dissolved in distilled water and/or other drugs. Concentrations of other drugs were 1 µM or 5 µM of NNGH, 0.25 µM or 0.5 µM of apocynin, 1 µM or 3 µM of GKT137831, 100 µM of antimycin A, 100 nM of Carbonyl cyanide-4-(trifluoromethoxy) phenylhydrazone (FCCP). Drugs were dissolved in DMSO.

### Lactate dehydrogenase assay and MTT reduction assay

The extent of cell death was assessed by using the cytotoxic assay kit to evaluate the activity of lactate dehydrogenase (LDH) released into the culture medium. Aliquots (50 µl) of cell-culture medium were incubated at room temperature in the presence of 0.26 mM NADH, 2.87 mM sodium pyruvate, and 100mM potassium phosphate buffer (pH 7.4) in a total volume of 200 µl, for 15–30 min. The levels of NAD^+^ formation were measured at 490 nm by using a microplate spectrophotometer (SPECTRA MAX 340 pc; Molecular Devices, Sunnyvale, CA). To assess cell viability, levels of MTT reduction were measured. After 24 h of 6-OHDA treatment, N27 cells were incubated with 0.5 mg/ml of MTT in medium overnight at 37°C. MTT is converted by viable cells to a water-insoluble precipitate that was dissolved in 10% SDS and colorimetrically quantified (O.D., 570 nm) by using a microplate spectrophotometer.

### Western blot analysis

Cells were washed with ice-cold PBS and lysed on ice in RIPA buffer (1% PBS, 1% NP-40, 0.5% sodium deoxycholate, 0.1% SDS) containing protease inhibitor mixture (AEBSF, aprotinin, bestatin hydrochloride, E-64-[N-(trans-epoxysuccinyl)-L-leucine 4-guanidinobutylamide], leupeptin, pepstatin A) (Sigma, Saint Louis, MO). A total of 30 µg of soluble protein per lane was loaded in SDS-PAGE and electrotransferred onto PVDF membrane. Specific protein bands were detected by using specific anti-Nox1 antibody (Santa Cruz Biotechnology, Santa Cruz, CA) and Enhanced Chemiluminescence (Pierce, Rockford, IL).

### Rac1 activation assay

Total cellular protein (500 µg) obtained from lysed N27 cells was incubated with 20 µl of agarose beads containing p21-binding domain (PBD) of p21-activated protein kinase 1 (PAK1), an effector of activated Rac, for 1 h at 4°C. The beads were collected by centrifugation and washed two times in the lysis buffer, resuspended in sample buffer. and boiled for 5 min. Proteins were resolved by SDS-PAGE using a 10%–20% Tricine gel, transferred electrophoretically and visualized using anti-rat Rac1 antibody, followed by electrochemoluminescence (ECL) detection. For the positive control, the nonhydrolyzable GTP analog GTPgS was used according to the manufacturer's protocol (Cell Biolabs, New York, NY).

### MMP3 activity assay

Matrix metalloproteinase-3 activity was measured using MMP-3 fluorescence assay kit following the manufacturer's instructions. To measure the activity of released MMP-3, 100 µL of the medium was collected and transferred to 96 wells, to which 99 µL of assay buffer (50 mM 2-(N-morpholino)ethanesulfonic acid, 10 mM CaCl_2_, and 0.05% Brij-35, pH 6.0) was added. To measure the activity of intracellular MMP-3, cells were washed in PBS and lysed by brief sonication in the assay buffer. About 50 µg of cell lysate protein was transferred to 96 wells, to which the assay buffer was added to a total volume of 199 µL. After incubation for 1 h at 37C, reaction was started by adding 1 µL (4 µL final concentration) of substrate (Mca-Pro-Leu-Gly-Leu-Dpa-Ala-Arg-NH2). The plates were read continuously in a fluorescence microplate reader (Molecular Devices) over 30 min at Ex/Em  = 328/393, and the rate of product formation was determined from the linear range.

### Nox activity assay

NADPH oxidase activity was measured by a lucigenin-derived chemiluminescence assay as described [21]–[23]. Briefly, 5–7 µg homogenized protein was incubated with its substrate NADPH (100 µM) in a phosphate buffer (50 mM, pH 7.0) containing 150 mM NaCl and 1 mM EGTA for 15 min, followed by an addition of 5 µM lucigenin for 15 min in dark. The chemiluminescent signal (photon emission) was measured using a Turner 20/20 luminometer (Turner Designs, Sunnyvale, CA). No activity could be measured in the absence of NADPH.

### Total RNA extraction and RT-PCR analysis

Total RNA was extracted from N27 cells using Trizol reagent (Invitrogen, Carlsbad, CA). Reverse transcription was performed for 1 h at 42°C with1 µg of total RNA using 20 unit/µl of AMV reverse transcriptase (Roche Applied Science, Indianapolis, IN), and oligo-p(dT)_15_ as a primer. The samples were then heated at 99°C for 5 min to terminate the reaction. The cDNA obtained from 1 µg total RNA was used as a template for PCR amplification. Oligonucleotide primers were designed based on Genebank entries for rat Nox1 (sense, 5′-TGAC AGTGATGTATGCAGCAT-3′; antisense, 5′-CAGCTTGTTGTGTGCACGCTG-3′), rat Nox2 (sense, 5′-ACTCGAAAACTTCTTGGGTCAG-3′; antisense, 5′-TCCTGTGATGCCAGCCAACCGAG-3′), rat Nox4 (sense, 5′-GCCGGCGGTATGGCGCTGTC-3′; antisense, 5′-CCACCATGCAGACACCTGTCAGG-3′), rat Noxa1 (sense, 5′-TCTAGGGGATCAGATACGGGAC-3′; antisense, 5′-CCAAGGAAATCCATGGGCTCCAG-3′), rat Noxo1 (sense, 5′-ACCCAGTATCAGCCCATGCTG-3′; antisense, 5′- ATGGAGCATCAGGAAGCTTGG-3′), rat p47 (sense, 5′-GTTAAAGGAGATGTTCCCCATTG-3′; antisense, 5′-TTATGAATGACCTCGATGGCTTC-3′), and rat GAPDH (sense, 5′-ATCACCATCTTCCAGGAGCG-3′; antisense, 5′-GATGGCATGGACTGTGGTCA-3′). PCR mixes contained 10 µl of 2X PCR buffer, 1.25 mM of each dNTP, 10 pmol of each forward and reverse primer, and 2.5 units of Taq polymerase in the final volume of 20 µl. Amplification was performed in 35 cycles at 60°C, 30 sec; 72°C, 1 min; 94°C, 30 sec. After the last cycle, all samples were incubated for an additional 10 min at 72°C for final extension step. PCR fragments were analyzed on 1.2% agarose gel in 0.5×TAE containing ethidium bromide. Amplification of GAPDH, a relatively invariant internal reference RNA, was performed in parallel, and cDNA amounts were normalized against GAPDH mRNA levels. The primer set specifically recognized only the gene of interest as indicated by amplification of a single band of expected size.

### Preparation and transfection of siRNA

Sense and anti-sense oligonucleotides corresponding to the following cDNA sequences of rat Nox1 (5′-CCTTTGCTTCCTTCTTGAAATCTAT-3′), rat MMP3 (5′ACGTACTTCTTTGTAGAGGACAAAT-3′ were used. The double-stranded siRNAs were synthesized chemically and modified into stealth siRNA (Invitrogen, Carlsbad, CA) to enhance the stability in vitro. Negative control stealth RNAi with a similar GC content as Nox1 stealth RNAi and MMP3 stealth RNAi was used. The sense and anti-sense oligonucleotides were annealed following the manufacturer's protocol to generate double-stranded siRNAs at the final concentration of 20 µM. N27 cells grown to 80% confluence in 6-well culture plates were subjected to transfection by adding 10 µl of Lipofectamin 2000 and 8 µl of 20 µM siRNAs (final concentration 40 nM). After 6 h of incubation, the culture medium was changed and cells were maintained for additional 30 h before analysis.

### Determination of cellular ROS content (MitoSox, DHE, and DCFDA) and mitochondrial membrane potential (TMRM)

ROS levels were measured by using three different methods: MitoSox, dihydroethidium (DHE), and Dichlorofluorescin diacetate (DCFDA) assays. In the MitoSox assay, MitoSOX Red mitochondrial superoxide indicator can be oxidized by superoxide (O_2_^• −^) and exhibits red fluorescence. In the DHE assay, blue fluorescent DHE can be dehydrogenated by superoxide (O_2_^• −^) to form a red fluorescent ethidium bromide. The DCFDA assay is based on the principle that the 20,70-dichlorodihydrofluorescein diacetate can be oxidized by ROS and converted to the fluorescent 20,70-dichlorofluorescein. Mitochondrial membrain potential (ΔΨ_m_) depolarization was measured by assessing changes in the fluorescence intensity of tetramethylrhodamine methyl ester (TMRM) accumulated in mitochondria within N27 cells. 6-OHDA-treated cells were incubated with MitoSox (5 µM), DHE (10 µM), DCFDA (100 µM), or TMRM (10 nM) in complete medium for 10 min at 37°C, respectively. To measure the fluorescence produced in the DCFDA and DHE, the medium was removed, and PBS was added to each well. The emitted fluorescence was read in a microplate spectrophotometer plate reader at Ex/Em 510/580 nm, Ex/Em 525/620 nm, Ex/Em 502/535 nm, or Ex/Em 543/575 nm for the MitoSox, DHE, DCFDA, or TMRM assays, respectively. MitoSox, DHE, and DCFDA were also determined on the confocal microscopy.

### Isolation of brain mitochondria

Five mice deeply anesthetized with sodium pentobarbital (120 mg/kg) and sacrificed. Mouse brain mitochondria were isolated by a modified isopycnic centrifugation procedure [24], employing Percoll density gradient. Briefly, cortex brain tissue was homogenized in the MSEGTA buffer comprising 225 mM mannitol, 75 mM sucrose, 5 mM HEPES, adjusted to pH 7.4, 1 mM EGTA and centrifuged at 1300×*g* for 3 min. The supernatant was collected and centrifuged at 21,000×*g* for 10 min. The pellet was resuspended in MSEGTA containing 15% (v/v) of Percoll and layered over discontinuous 24%(v/v), 40% (v/v) Percoll/MSEGTA and centrifuged at 30,700×*g* for 10 min. Purified mitochondria fraction was collected from the top of the 40% to the middle of 24% Percoll layer of the tube, resuspended in MSEGTA and washed 2 times by centrifuging at 20,000×g for 10 min. Final mitochondrial pellet was resuspended in MS buffer comprising 250 mM mannitol, 75 mM sucrose, 4 mM KH2PO4, 20 mM HEPES, pH 7.2 and stored on ice. Protein content was estimated by a commercial BCA assay (BioRad, Hercules, CA, USA). Mitochondirial purity was determined by Western blot analysis against cytosolic tubulin, nuclear Histone H3, and mitochondiral Tim 23 antibodies.

### Measurement of H_2_O_2_ in mitochondrial fraction

H_2_O_2_ was measured using Amplex Red with Horse radish peroxidase (HRP) by the following reaction: Amplex Red + H_2_O_2_ -> resorufin + O_2_. Resorufin is a stable and highly fluorescent compound, where we measured at excitation of 571 nm and at emission of 585 nm. The fluorescence of resorufin was determined in standard black 96-well-plates in incubation medium consisted of 225 mM sucrose, 75 mM mannitol, 1mM EGTA, and 5 mM HEPES (pH 7.4) with 2 mM glutamate and 2 mM malate as respiratory substrate plus recombinant CD MMP3 (rCD MMP3), rCD MMP3 and NNGH or rotenone, 0.05 mg of mitochondrial protein per well, 0.2 U/ml HRP and 50 µM Amplex Red. Fluorimetric measurements were made using fluorimeter SpectraMax M5 (Molecular Devices). Rotenone was used as a positive control. A standard curve of known concentrations of H_2_O_2_ was used for determining the molar concentration of H_2_O_2_ generated by rCD MMP3.

### Expression of autoactivation mutation MMP3 and inactive mutation of MMP3

Human MMP3 full length was inserted between EcoRI and XhoI sites in the pAAV-IRES-hrGFP vector (Invitrogen, Carlsbad, CA, USA). Autoactivation mutation form (AutoMMP3), the point mutants of MMP3 (Val to Gly) in the residues 94 were prepared using the QuickChange site Mutagenesis Kit (Agilent Technologies Inc., Santa Clara, CA, USA). Wild type of MMP3 in pcDNA3.1(Invitrogen, Carlsbad, CA, USA) was used as a template with the sense oligo 5′- GTGTG GAGGTCCTGA TGTTGG -3′ and anti-sense oligo 5′- CCAACATCAGGACCTCCACAC-3′ to generate the autoactivation mutant. Inactive mutant MMP-3 was generated by substitution of glutamate at amino acid 219 with alanine (Glu to Ala) by site-directed mutagenesis using PCR. All constructs were confirmed by sequencing. DNA transfections were performed using Lipofectamine 2000 (Invitrogen, Carlsbad, CA, USA), following the manufacturer's guidelines.

### *Mmp3* KO mice and MPTP injection

This study was carried out in strict accordance with the recommendations in the Guide for the Care and Use of Laboratory Animal Research Center of Konkuk University. All procedures were approved by the Animal Experiment Review Board of Laboratory Animal Research Center of Konkuk University (Permit Number: KU 12035). *Mmp3* null mice (C57BL/6x129SvEv) were generously donated by Dr. Onyou Hwang (University of Ulsan of College of Medicine). *Mmp3* knockout (KO) mice (C57BL/6x129SvEv), originally developed by Mudgett et al. [25], and their wild-type (WT) animals were obtained from Taconic Farms (Germantown, NY) and bred at the specific pathogen free animal facility of Asan Institute for Life Science, University of Ulsan of College of Medicine (Seoul, Korea). WT and *Mmp3* null mice (male, 8 weeks, body weight 20±2 g) received 4 intraperitoneal (i.p.) injections of PBS or MPTP (4×15 mg/kg at 2 h intervals). After 7 days, mice deeply anesthetized with sodium pentobarbital (120 mg/kg) and were sacrificed (5 mice per group).

### Stereotaxic injection of AAV2 particles

The experiments were carried out on rats, in accordance with the National Institutes of Health Guide for the Care and Use of Laboratory Animals. All procedures were approved by the Institutional Animal Care and Use Committee (IACUC) of Weill Cornell Medical College (Permit Number: 0709-667A). 68 male Sprague Dawley (SD) rats (Charles River; 8-weeks-old at the time of the beginning of AAV expression, 2 or 3 per cage) were maintained in a temperature/humidity-controlled environment under a 12 h light/dark cycle with free access to food and water. All rats were respectively allocated into 2 groups, Nox1 shRNA/AAV, T17N Rac1/AAV, and scramble (scb) shRNA/AAV or empty vector/AAV as control groups. All surgery was performed under ketamine (50 mg/kg) and Xylazine (5 mg/kg) mixture (i.p.) anesthesia, and all efforts were made to minimize suffering. Rats placed in a rat stereotaxic apparatus, a site in the right substantia nigra (SN) [coordinate: anteroposterior (AP), −5.3 mm; mediolateral (ML), +2.0 mm; dorsoventral (DV), −5.8 mm] was selected to inject scb shRNA/AAV (n = 8), Nox1 shRNA/AAV (n = 16), T17N Rac1/AAV (n = 16), and empty vector/AAV (n = 28), respectively, according to the grouping. A total of 1×10^11^ genome copy/ml rAAV particles encoding shNox1, T17N rac1, scb, or empty vector diluted in 2 µl ice-cold sterilized phosphate buffered saline (PBS) were used in every animal. Four animals of Nox1 shRNA/AAV, T17N Rac1/AAV, or empty vector group were used for the detection of Nox1 shRNA, T17N Rac1, or empty vector expression at 4 weeks following AAV particle injection. Eighteen animals received ipsilateral injection of autoactivated MMP3/AAV while all other rats received ipsilateral injection of inactive mutant MMP3/AAV a site in the right SN. The injection rate was 0.5 µl/min, and the syringe was kept in place for an additional 5 min before being retracted slowly. After 1, 2, and 3 weeks, rats deeply anesthetized with sodium pentobarbital (120 mg/kg) and sacrificed (6 rats per group).

### Immunohistochemistry

Rats were deeply anesthetized with sodium pentobarbital (120 mg/kg) and transcardially perfused with saline containing 0.5% sodium nitrite and 10 U/ml heparin sulfate, followed by 4% cold formaldehyde generated from paraformaldehyde in 0.1 M PBS (pH 7.2). Brains were post-fixed in the same solution for overnight and infiltrated with 30% sucrose overnight. Free-floating sections (40 µm) were obtained from the striata and SN using a Cryostat. Sections were washed in 0.1 M PBS, incubated in 0.1 M PBS containing 5% normal goat serum and 0.3% TritonX-100 for 1 h, and subsequently incubated overnight with Nox1 (1∶500), or TH (1∶10,000), antibodies at 4°C. The sections were then incubated with appropriate secondary IgG (1∶200) for 1 h, followed by avidin/biotin/peroxidase staining for 1 h in a humidified chamber. Washing of the sections on slides was done using 0.1 M PBS containing 1.5% bovine serum albumin. The antigen–antibody complexes were visualized by incubation for 5 min in 0.05% 3,3′-diaminobenzidine and 0.003% H_2_O_2_. Mounted slides rinsed quickly in distilled water, and dehydrated in serially diluted ethanol, and cleaned in xylene followed by sequential mounting in glass slides using permanent mounting medium. Mounted slices were evaluated on light microscope.

### Fluorescent immunostaining of tissues

Free-floating sections (40 µm) were washed in 0.1 M PBS, incubated in 0.1 M PBS containing 5% normal donkey serum and 0.3% TritonX-100 for 1 h, and subsequently incubated overnight with primary antibodies (TH, 1∶2,000, MMP3, 1∶500, Nox1, 1∶200, and MDA 1∶500) in 2% normal donkey serum in PBS at 4°C and incubated in a 1∶200 dilution of Alexa Fluor conjugated donkey anti-rabbit (546 or 647), donkey anti-mouse (488 or 546), or donkey anti-goat (488) antibodies for 1 h at room temperature, washed with PBS, and then mounted sequentially in glass slides using Vectashield. Mounted slices were evaluated for fluorescence under settings for 546, 488, and 660 nm emissions on a confocal microscope (Leica TCS SP5).

### TH immunostaining and TH- positive cells counting

A set consisting of six sections, 360 µm apart, were prepared. Sections were used for tyrosine hydroxylase (TH) immunohistochemistry using avidin–biotin peroxidase technique (Vectastain ABC kit from Vector Labs, Burlingame, CA). A rabbit anti-TH affinity purified antibody (1: 10,000; from Protos Biotech, New York, NY) was used. Numbers of TH-immunoreactive cells in the substantia nigra (SN) were counted using the optical fractionator [26]. Analysis was performed using a system consisting of a Nikon Eclipse E600 microscope (Morrell Instruments Co. Inc., Melville, NY) equipped with a computer-controlled LEP BioPoint motorized stage (Ludl Electronic Products, Hawthorne, NY), a DEI-750 video camera (Meyer Instruments, Houston, TX), a Dell Dimension 4300 computer (Dell, Round Rock, TX), and the Stereo Investigator (v. 4.35) software program (Microbrightfield, Burlington, VT). Tissue sections were examined using a Nikon Plan Apo 100·objective lens with a 1.4 numerical aperture. The size of the x–y sampling grid was 140 µm. The counting frame thickness was 30 µm and the counting frame area was 4900 µm^2^. The coefficient of error and coefficient of variation were also determined.

### Quantitative analysis

Sections including the cortex and striatum from four rats per group were subjected to analysis. Five regions of interests (ROIs) of 0.1 mm^2^ per one section in the SN areas (bregma −4.8 to −6.0 mm; 6 sections per rat, every fifth sections) were selected. The number of Nox1-, or MDA-positive cells were counted in each ROI and averaged. Data represented as percentage of total cell. All quantitative analyses were carried out in a blind manner.

### Statistical analysis

Data are expressed as percentages of values obtained in control conditions and are presented as mean ± S.E.M. of at least three experiments in independent cell cultures. Statistical analysis was performed using a one-way ANOVA followed by Newman-Keuls Multiple Comparison Test. Values of *p<*0.05 were considered significant.

## Results

### Nox1 and MMP3 expressions and activities are increased in dopaminergic neuronal cells treated with 6-OHDA

**Figure 1:**
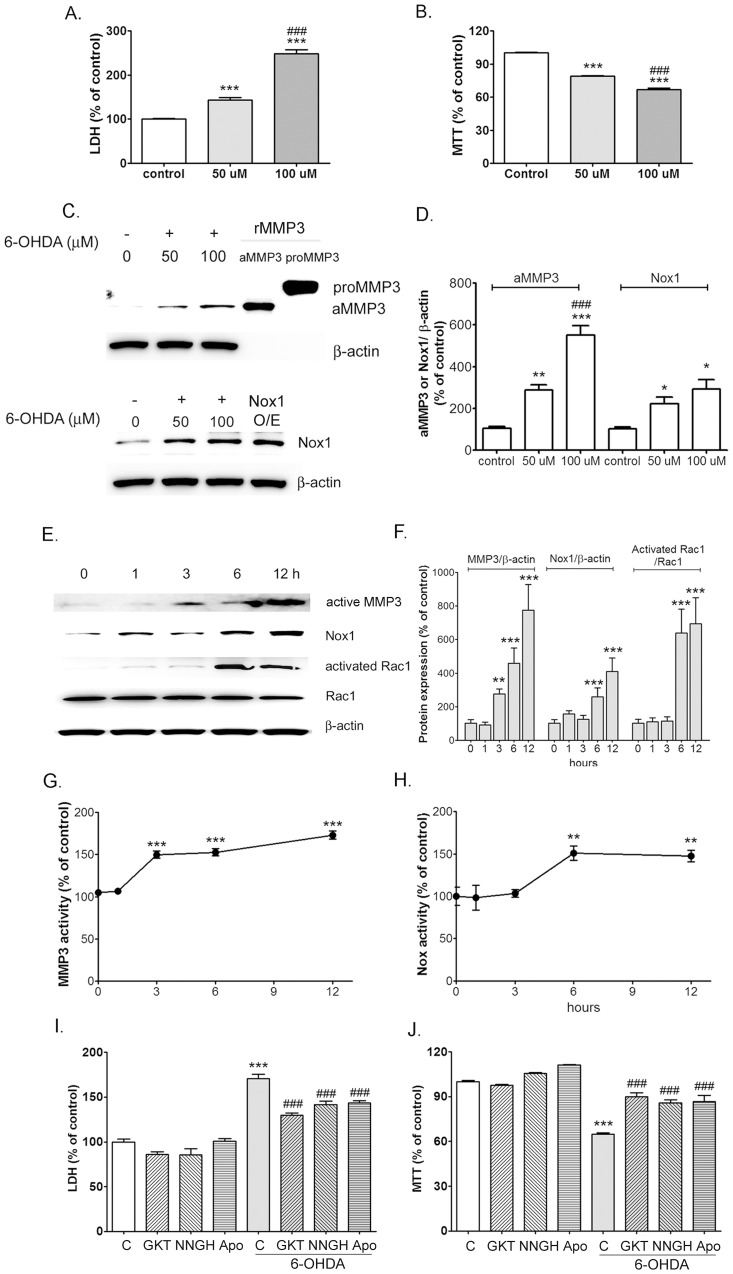
MMP3 activation and Nox1 activation are responsible for 6-OHDA-mediated cell death in N27 dopaminergic cells. (A and B) 6-OHDA-mediated cell death was measured at 24 h post treatment by the LDH (A) and MTT (B) assay. (C) Representative photomicrographs of Western blots for MMP3, Nox1, and β-actin at 24 h after 6-OHDA treatment in the N27 cells. rMMP3, recombinant MMP3 proteins; proMMP3, preform of rMMP3; aMMP3, active form of rMMP3, Nox1 O/E; lysates of N27 cell overexpressed human full length of Nox1 (D) Signal intensities were measured by Quantity One software and shown as a % of control. β-actin, internal control; **p<0.01, ***p<0.001 vs. non-treated control; ###p<0.001 vs. 50 uM 6-OHDA treated cells. (E) Representative photomicrographs of Western blots for active MMP3, Nox1, activated Rac1, and Rac1 at 1, 3, 6, 12 h after 6-OHDA treatment in the N27 cells. (F) Signal intensities were measured by Quantity One software and shown as a % of control. β-actin or Rac1, internal control. ** p<0.01, ***p<0.001 vs. non-treated control. (G) MMP3 enzyme activity and (H) Nox enzyme activity were measured in lysates of N27 cells at 1, 3, 6, 12 h after 100 µM 6-OHDA treatment. ** p<0.01, ***p<0.001 vs. non-treated control. (I and J) NNGH, Apocynin, and GKT reduced cell death as determined the LDH (I) and MTT (J) assay at 24 h post 6-OHDA (100 µM). ***p<0.001 vs. non-treated control; ###p<0.001 vs. 6-OHDA control. GKT: GKT137831, a Nox1 selective inhibitor. Results are presented as the mean ± SEM, n = 8. The whole experiment has been repeated four times with similar results.

To evaluate the effects of 6-OHDA treatment on MMP3 and Nox1 expression and activation, N27, a well-established rat DA cell line was examined [20]. 6-OHDA showed a concentration-dependent cytotoxicity in N27 cells (Fig. 1A and 1B). We also observed that 6-OHDA increased active form of MMP3 in a dose-dependent manner (Fig. 1C and 1D). Similarly, low basal level of Nox1 was highly elevated by 6-OHDA treatment, as shown by Western blot analyses (Fig. 1C and 1D). To confirm the results of the MMP3 and Nox1 immunoblotting analysis, recombinant proMMP3, recombinant acive form of MMP3, and total lysates of N27 cells overexpressed human full lengh of Nox1 were used as a positive control, respectively (Fig. 1C). The expressions of MMP3 and Nox1 were measured in DA cells treated with 6-OHDA for various duration from 1 h to 12 h. After challenging with 6-OHDA, active form of MMP3 expression in N27 cells started to increase from 3 h. On the other hand, Nox1 expression was increased from 6 h (Fig. 1E and 1F). The activation of a small GTPase Rac1 is indispensable for Nox1 and Nox2 activation [27]. GTP-bound active Rac1 was measured in DA cells treated with 6-OHDA for various duration from 1 h to 12 h. Increased Rac1 activity was observed at 6 h post 6-OHDA administration (Fig. 1E and 1F). Intracellular MMP3 and Nox activities were also determined from 1 h to 12 h post 6-OHDA administration. We observed that intracellular MMP3 and Nox activation were significantly elevated at 3 h and 6 h post 6-OHDA administration, respectively (Fig. 1G and 1H). These results indicate that MMP3 activation is prior to Nox1 expression and activation in DA cells after 6-OHDA treatment. To clarify the specific role of MMP3 and NADPH oxidase in 6-OHDA mediated DA cell death, we tested the effect of inhibition of MMP3 and Nox1 on DA cell death. The effects of NNGH, an MMP3 inhibitor, apocynin, a putative inhibitor of NADPH oxidase activity and GKT137831, a Nox1 and Nox4 inhibitor on 6-OHDA-mediated DA cell death were tested. Cultures were pretreated with 5 µM NNGH, 500 nM apocynin, 1 or 3 µM GKT137831 for 1 h and then treated with 100 µM 6-OHDA. The viability of cells was determined with LDH and MTT assays. NNGH, apocynin, and GKT137831 significantly reduced LDH levels by 28.9%, 27.1%, and 41.0% in cultures treated with 100 µM 6-OHDA, respectively, compared with cultures treated with 6-OHDA alone (Fig. 1I). The levels of reduced MTT were statistically higher in cultures pretreated with NNGH, apocynin, and GKT137831 than in those treated with 6-OHDA only. As shown in Fig. 1J, NNGH, apocynin, and GKT137831 increased the levels of reduced MTT by 21.0%, 21.8%, and 25.2% in cultures treated with 100 µM 6-OHDA, respectively. Overall, the results indicate that MMP3 and Nox1 may be a key player in 6-OHDA -mediated dopaminergic neuronal toxicity.

### MMP3 regulates the expression of Nox1 in dopaminergic neuronal cells treated with 6-OHDA

**Figure 2:**
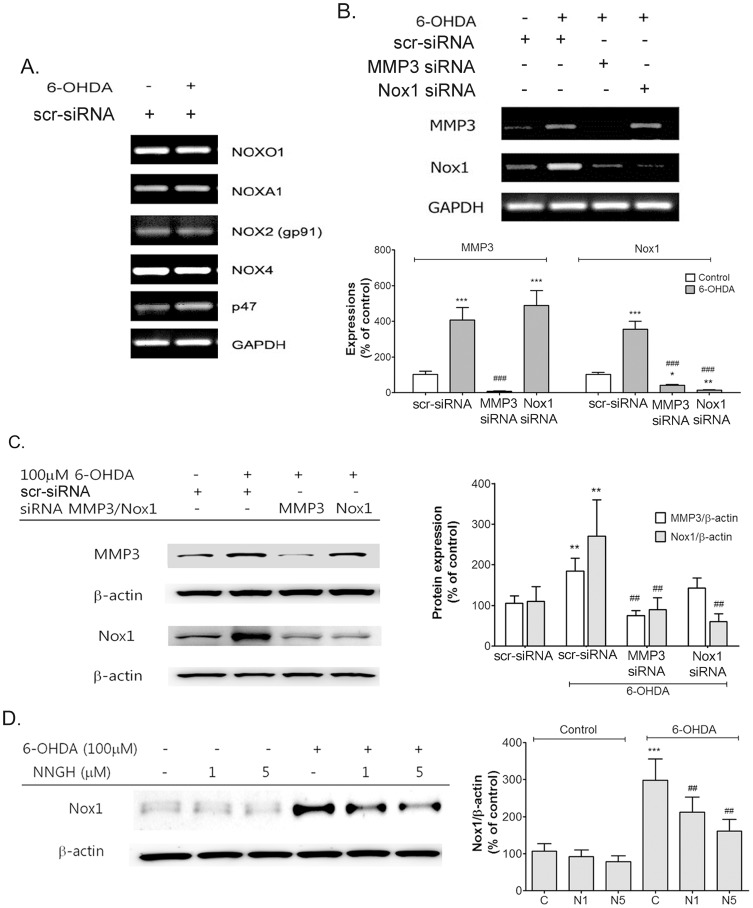
MMP3 regulate the expression of Nox1 in 6-OHDA treated N27 cells. (A) A representative RT-PCR performed on various Nox subunits from RNA preparation of N27 cells treated with 100 µM 6-OHDA. (B) A representative RT-PCR performed on MMP3 and Nox1 from RNA preparation of N27 cells. N27 cells were transfected with two different siRNAs against MMP-3 and Nox1 and their scrambled sequences (scr-siRNA) and subsequently treated with 6-OHDA for 6 h. Densitometric analysis of the RT-PCR results normalized against GAPDH and expressed as % induction compared with respective untreated control. * p<0.05, ** p<0.01, ***p <0.001 vs. control ; ###p<0.001 vs. 6-OHDA control. (C) Representative photographs of western blot for MMP3 and Nox1 on lysate of N27 cells transfected with two different siRNAs against MMP-3 and Nox1 and scr-siRNA and subsequently treated with 6-OHDA for 24 h. Densitometric analysis of the Western blot results normalized against β-actin and expressed as % induction compared with respective untreated control. ** p<0.01 vs. scr-siRNA control ; ##p<0.01 vs. scr-siRNA+6-OHDA control. (D) A representative western blot against Nox1 on lysate of N27 cells pre-treated with 1 µM and 5 µM N-isobutyl-N-(4-methoxyphenylsulfonyl) glycyl hydroxamic acid (NNGH) for 30 min followed by exposure to 100 µM 6-OHDA for 24 h. Densitometric analysis of the western blots results normalized against β-actin and expressed as % expression compared with respective untreated control. ***p<0.001 vs. control ; ##p<0.01vs. 6-OHDA control. Results are presented as the mean ± SEM, n = 8. The whole experiment has been repeated four times with similar results.

Nox isoforms (Nox2, and Nox4) and Nox regulators (Noxo1, Noxa1, and p47) were detected constitutively in N27 cells in the presence or absent of 6-OHDA (Fig. 2A). MMP3 and Nox1 was induced by 100 µM 6-OHDA treatment for 6 h in N27 cells (Fig. 2B). To evaluate the effect of MMP3 in Nox1 induction on DA cell death, MMP3 and Nox1 knockdown were achieved by RNAi. MMP3 or Nox1 knockdown efficiency of each siRNA nucleotide sequence was assessed at 6 h and 24 h post 6-OHDA treatment by both RT-PCR (Fig. 2B) and immunoblotting (Fig. 2C) following 36 h post transfection . Interestingly Nox1 induction was inhibited by MMP3 knockdown. However, MMP3 induction was not changed by Nox1 knockdown (Fig. 2B). MMP3 protein expression was blocked by MMP3 siRNA transfection in 6-OHDA treated N27 cells and Nox1protein expression was decreased by MMP3 knockdown in N27 cells after treatment with 6-OHDA. However, 6-OHDA-induced MMP3 protein expression was not altered by Nox1 knockdown (Fig. 2C). Next, in the presence of the MMP3 inhibitor NNGH, Nox1 protein expression was tested by Western blot analysis in N27 cells treated with 6-OHDA . Nox1 expression level was decreased in a NNGH dose-dependent manner in DA neuronal cell treated with 6-OHDA (Fig. 2D). These results suggest that MMP3 can control the Nox1 expression in 6-OHDA treated DA neuronal cells.

### MMP3-induced mitochondrial ROS regulate the Nox1 expression in dopamanergic neuronal cells treated with 6-OHDA

**Figure 3:**
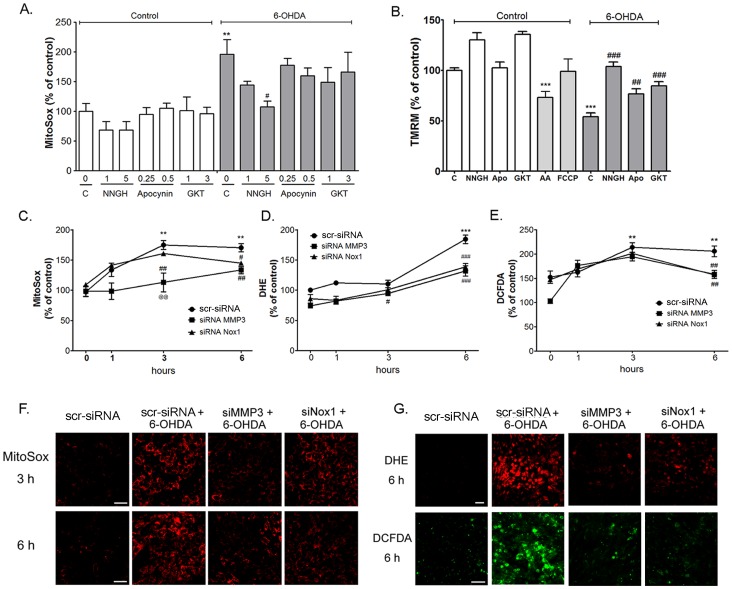
MMP3 induced mitochondrial ROS regulate the Nox1 expression in 6-OHDA treated N27 cells. (A) Mitochondrial ROS generation was measured using the MitoSox staining in N27 dopaminergic cells treated with post 6-OHDA (100 µM) for 1 h. NNGH significantly reduced 6-OHDA-mediated ROS generation. (B) Mitochondrial membrane potential (ΔΨm was measured using the TMRM staining in N27 dopaminergic cells treated with post 6-OHDA (100 µM) for 6 h. NNGH, apocynin, and GKT significantly inhibited decreasing of 6-OHDA-mediated ΔΨm. Apo: apocynin, a Nox inhibitor; GKT: GKT137831, a Nox1 selective inhibitor; AA, antimycin A (100 µM); FCCP, an oxidative phosphorylation uncoupler (100 nM), Results are presented as the mean ± SEM, n = 8. ** p<0.01, ***p<0.001 vs. nontreated control; # p<0.05, ## p<0.01, ### p<0.001 vs. 6-OHDA control. (C to E) ROS level was measured using the MitoSox (C), DHE (D), and DCFDA (E) in N27 cells transiently transfected with rat Nox1 siRNA, MMP3 siRNA or control siRNA for 36 h, followed by 6-OHDA exposure for 1, 3, and 6 h. Results are presented as the mean ± SEM, n = 8. The whole experiment has been repeated four times with similar results. ** p<0.01, ***p<0.001 vs. nontreated control at 0 h; # p<0.05, ## p<0.01, ### p<0.001 vs. 6-OHDA control at 3 or 6 h. @@ p<0.01 vs. siRNA Nox1 treated with 6-OHDA at 3 h. (E to F) Representative photomicrograph of the MitoSox (E), DHE (F), and DCFDA (F) staining showing ROS levels in N27 cells transiently transfected with rat Nox1 siRNA, MMP3 siRNA or control siRNA for 36 h, followed by 6-OHDA exposure for 3, 6, or 9 h. Scale bar  = 50 µm.

**Figure 4:**
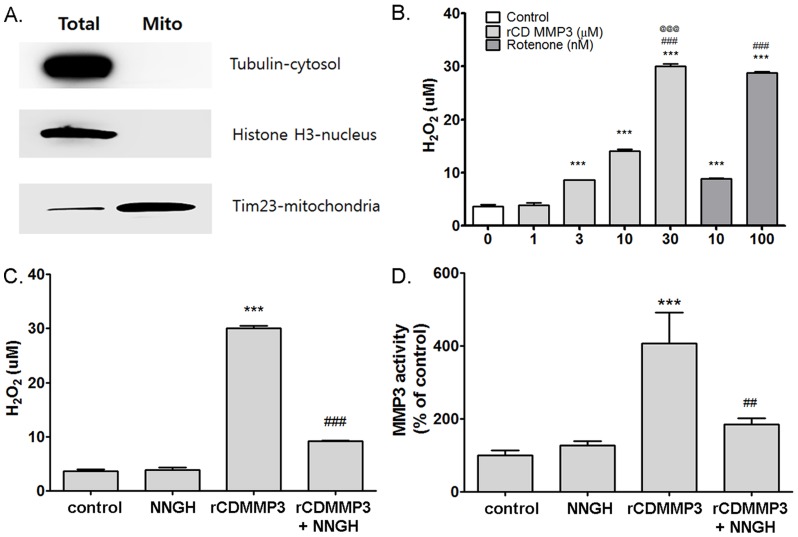
Recombinant catalytic domain of MMP3 (rCD MMP3) induced mitochondrial ROS generation in mitochondrial fraction. (A) Representive photographs of Western blots for tubulin, histone H3, Tim 23 in the total lysates and mitochondrial fractions of the brain tissues. (B) Mitochondrial H_2_O_2_ levels were determined by the Amplex red assay. H_2_O_2_ levels after incubation with 1, 3, 10, and 30 µM of rCD MMP3 or 10 and 100 nM of rotenone in mitochondrial fraction for 1 h. *** p<0.001 vs. control, ### p<0.001 vs. 3 µM of rCD MMP3 or 10 nM of Rotenone, @@@ p<0.001 vs. 10 µM of rCD MMP3. (C) H_2_O_2_ levels after incubation with 30 µM of rCD MMP3 in the absence or presence of NNGH (30 µM) for 1 h. *** p<0.01 vs. control, ### p<0.001 vs. rCD MMP3. (D) MMP3 activity was measured in mitochondrial fraction after rCD MMP3 (30 µM) incubation in the absence or presence of NNGH (30 uM) for 1 h. *** p<0.01 vs. control, ## p<0.001 vs. rCD MMP3.

**Figure 5:**
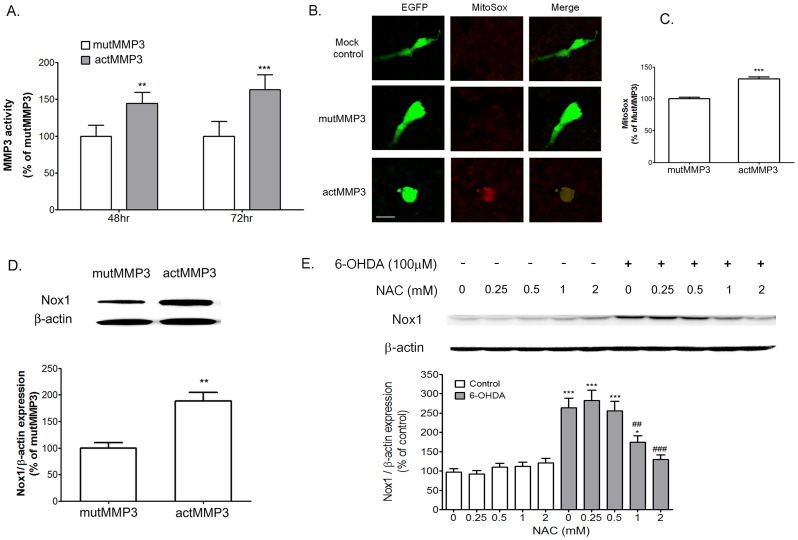
Autoactivated MMP3 (actMMP3) induced mitochondrial ROS generation and the Nox1 expression. N27 cells were transfected with mock control, catalytically inactive mutant MMP3 (MutMMP3), or actMMP3 vector for 24, 48, or 72 h. Mitochondrial ROS levels were determined by the MitoSox staining (A) and MitoSox level (B) was quantified at 24 h after transfected with MutMMP3 and ActMMP3. Cells expressing GFP (green) represent vector-transduced cells. Scale bar  = 10 µm (C) MMP-3 enzyme activity assay on lysates of N27 cells transfected with MutMMP3 and ActMMP3 for 48 or 72 h. (D) Representative photomicrographs of Western blots against Nox1 on lysates of N27 cells transfected with MutMMP3 and ActMMP3 for 48 h. Densitometric analysis of the western blots results normalized against β-actin and expressed as % induction compared with MutMMP3-transfected cells. Results are presented as the mean ± SEM, n = 8. The whole experiment has been repeated three times with similar results. ** p<0.01, *** p<0.001 vs. cells transfected with MutMMP3 vector. (E) Representative photomicrographs of Western blots against Nox1 on lysates of N27 cells treated with 0.25, 0.5, 1, 2 mM of N-acetylcystein (NAC) in the presense or absence of 6-OHDA. Cells were cotreated with 6-OHDA and NAC for 24 h. Densitometric analysis of the western blots results normalized against β-actin and expressed as % induction compared with non-treated control cells. Results are presented as the mean ± SEM, n = 4. The whole experiment has been repeated three times with similar results. * p<0.05, *** p<0.001 vs. non-treated control cells; ## p<0.01, ### p<0.001 vs. 6-OHDA treated cells.

Mitochondria play a key role in Nox1-mediated superoxide generation [14]–[16]. We previously reported that mitochondrial respiratory complex inhibitors which increased mitochondrial ROS also induced Nox1 in N27 cells, suggesting a role of mitochondrial ROS in Nox1 induction [13]. To clarify the specific role of MMP3 in 6-OHDA-mediated mitochondrial ROS generation, we tested the effect of NNGH, apocynin, and GKT137831 in N27 cells treated with 6-OHDA. N27 cells were pretreated with 1 µM and 5 µM NNGH, 0.25 µM and 0.5 µM apocynin, and 1 µM and 3 µM GKT137831 for 1 h, and then 100 µM of 6-OHDA was added. Mitochondrial ROS levels were measured at 1 h after 6-OHDA by using a MitoSox assay. Interestingly while 6-OHDA-induced mitochondrial ROS level was significantly decreased by 5 µM of NNGH, mitochondrial ROS level was not changed by apocynin and GKT137831 (Fig. 3A). We also checked whether NNGH, apocynin and GKT137831 can affect 6-OHDA-mediated change in mitochondrial membrain potential (ΔΨ_m_) by assessing changes in the fluorescence intensity of TMRM accumulated in mitochondria within N27 cells. (Fig. 3B). ΔΨ_m_ levels were measured in N27 cells at 6 h after 6-OHDA treatment. N27 cells treated with 6-OHDA reduced a 45.94±3.92% in fluorescence intensity of TMRM (n = 8, Fig. 3B). 6-OHDA-induced decrease in TMRM fluorescence in N27 cells was reversed by NNGH, apocynin or GKT137831 (p<0.001, Fig. 3B). Antimycin A (100 µM) and FCCP (100 nM) were used as a positive control and a negative control, respectivly. NNGH, apocynin and GKT137831 in normal culture condition did not affect mitochondrial ROS generation and membrane potentials at the similar doses. For further verification, we tested the effect of inhibition of MMP3 expression or Nox1 expression on mitochondrial ROS by employing the siRNA knockdown technique. ROS levels of N27 cells were measured by microreader and confocal microscope (Fig. 3C∼3G). Cells transfected with scrambled sequences showed increased levels of mitochondrial ROS, cellular superoxide, and cellular ROS in N27 cells treated with 6-OHDA which were determined by MitoSox, DHE and DCFDA, respectively (Figs. 3C, 3D, 3F, and 3G). MMP3 knockdown significantly reduced MitoSox and DHE levels at 3 and 6 h after 6-OHDA treatment, respectively (Figs. 3C, 3D, 3F, and 3G). Nox1 knockdown decreased MitoSox and DHE levels at 6 h after 6-OHDA treatment (Figs. 3C, 3D, 3F, and 3G). DCFDA levels were inhibited by MMP3 and Nox1 knockdown at 6 h after 6-OHDA treatment (Fig. 3E and 3G). These results suggested that MMP3 activation might increase mitochondrial ROS at 3 h after 6-OHDA treatment. Next, to verify direct effect of MMP3 on mitochondrial ROS generation, we tested whether active MMP3 can directly induce ROS generation from the isolated mitochondria (Fig. 4A). Generation of H_2_O_2_ was determined in the presence of rCD MMP3 in the purified mitochondria. Rotenone (10 nM and 100 nM) was used as a positive control. rCD MMP3 significantly increased mitochondiral H_2_O_2_ generation in a dose-dependent manner (Fig. 4B). However, NNGH treatment reversed rCD MMP3-induced H_2_O_2_ production (Fig. 4C). NNGH-mediated inhibition of MMP3 activity significantly reduced mitochondiral H_2_O_2_ generation, confirming that active MMP3 can directly induce mitochondrial ROS. (Fig. 4D). Next, we tested whether MMP3-induced mitochondrial ROS cause the expression of Nox1 in DA neurons. To prove the effect of MMP3 on the expression of Nox1, we overexpressed EGFP-tagged autoactivated MMP3 (actMMP3) or catalytically inactive mutant MMP3 (mutMMP3) in N27 cells. MMP3 activity assay, MitoSox assay, and Western blot analysis were performed to prove the interplay between mitochondrial ROS and Nox1 expression caused by actMMP3 at various time points after transfection (24, 48, and 72 h). Overexpression of actMMP3 increased intracellular MMP3 activity in N27 cells at 48 h and 72 h after transfection (Fig. 5A). At such condition, mitochondrial ROS and Nox1 expression were significantly increased (Fig. 5B, 5C, and 5D), suggesting that actMMP3 may induce Nox1 expression via generation of mitochondrial ROS in DA neuronal cells challenged with 6-OHDA. We also confirmed that Nox1 expression in the 6-OHDA treated N27 cells reduced in the presense of N-acetylcysteine (NAC), a ROS scavenger, in a dose dependent manner (Fig. 5 E).

### Nox1 expression is abolished in MMP3 knockout mice treated with MPTP

**Figure 6:**
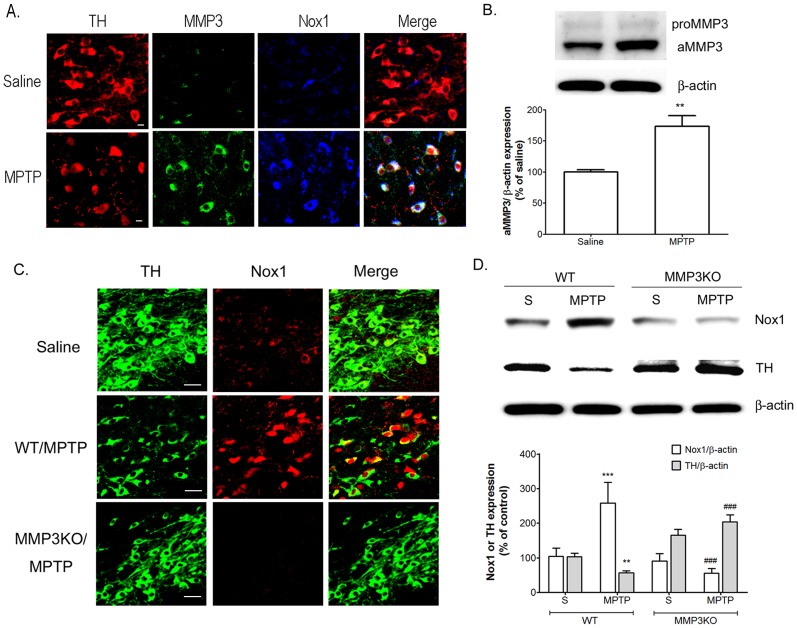
Nox1 expression abolished MPTP treated MMP3 knockout mice. (A) MMP3 and Nox1 expression was increased in the rat SN DA neurons after MPTP administration. TH (red), MMP3 (green), and Nox1 (blue) were visualized in the mouse SN at 7 days post-i.p. injection of vehicle or MPTP. MMP3 and Nox1 coexpression in TH^+^ DA neurons is demonstrated as white staining after merging red (TH), green (MMP3) and blue (Nox1) images. Scale bars  = 10 µm. (B) Representative photomicrographs of Western blots against MMP3 on lysates of SN tissue at 7 days post-i.p. injection of MPTP or vehicle. Densitometric analysis of the western blots results normalized against β-actin and expressed as % expression compared with vehicle injected rat. (C) Nox1 expression was increased in the rat SN DA neurons after MPTP administration in wild type mouse (WT) not in MMP3 knockout mouse (MMP3KO). TH (green) and Nox1 (red) were visualized in the SN at 7 days post-i.p. injection of MPTP in WT (middle panels), MMP3 KO (lower panels) or vehicle in WT (upper panels). Nox1 expression in TH^+^ DA neurons is demonstrated as yellow staining after merging green (TH) and red (MMP3) images. Scale bars  = 20 µm. (D) Representative photomicrographs of Western blots against Nox1 and TH on lysates of SN tissue at 7 days post-i.p. injection of MPTP or vehicle in WT or MMP3KO mice. Densitometric analysis of the western blots results normalized against β-actin and expressed as % expression compared with vehicle injected WT rat. Results are presented as the mean ± SEM, n = 5. ** p<0.01, *** p<0.001 vs. saline treated rats, ### p<0.001 vs. MPTP treated rats.

We previously reported that significant attenuation of MPTP-elicited SN DA neuronal degeneration in the absence of MMP3. To verify the effect of MMP3 on Nox1 expression in SN DA neurons of MPTP-injected mice, wild type mice and *Mmp3* knockout mice were subjected to MPTP i.p. injection. MMP3 and Nox1 expressions were increased and colocalized in SN DA neurons at 7 days post MPTP treatment (Figs. 6A and 6B). While Nox1 expression was dramatically increased in wild type mice, MPTP failed to increase Nox1 expression in SN DA neurons of *Mmp3* KO mice (Figs. 6C and 6D).

### MMP3 causes SN DA neurodegeneration via increased Nox1 expression *in vivo*

**Figure 7:**
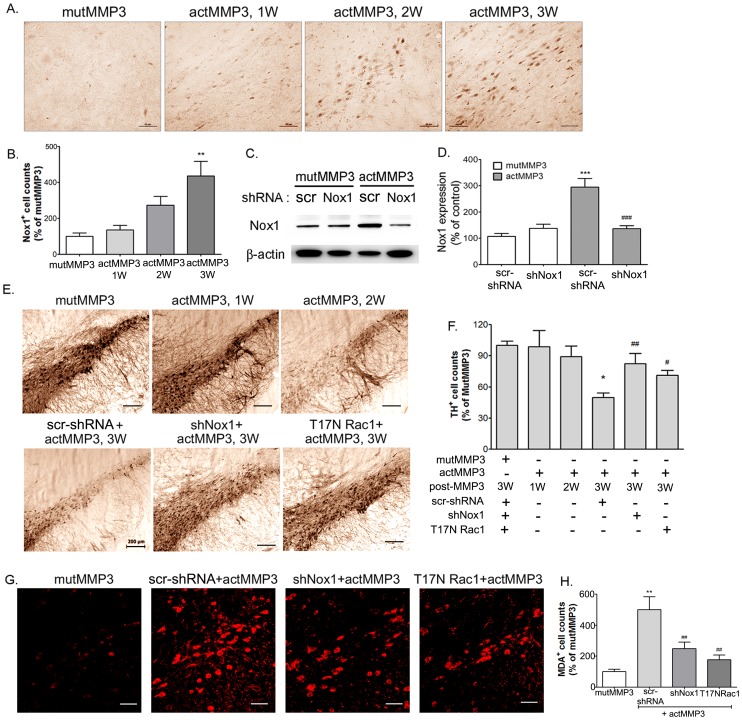
Increase in Nox1 protein levels in the SN of rats injected with autoactivated MMP3 (actMMP3). (A) Representative photomicrographs of Nox1 -immunoreactivity in the SN sections. AAV2 particles containing mutMMP3 or actMMP3 were stereotaxically injected into the rat SN. After 1, 2, 3 weeks later, DA neurons in the SN were visualized with Nox1 immunostaining. Scale bar  = 50 µm. (B) Stereologic counts of Nox1-positive neurons in the SN shown as percentage of mutMMP3. ** p<0.01 vs. mutMMP3 injected rats. (C and D) Nox1 knockdown efficiency in the SN was verified by western blot analysis performed at 7 weeks after AAV2 injection. n = 4/group, *** p<0.001 vs. shNC and mutMMP3 injected rats. ### p<0.001 vs. shNC and actMMP3 injected rats. shNC, scramble RNA/AAV2. (E) Representative photomicrographs of TH staining in the rat SN sections. To knockdown Nox1 in the SN, AAV2 particles harboring Nox1 shRNA (shNox1) or T17N Rac1 were stereotaxically injected into the SN. After 4 weeks incubation, AAV2 particles containing mutMMP3 or actMMP3 were stereotaxically injected into the rat SN. Three weeks later, DA neurons in the SN were visualized with TH immunostaining. Scale bar  = 200 µm (F) Stereologic counts of TH-positive neurons in the SN shown as percentage of mutMMP3. Results are presented as the mean ± SEM. n = 6–7. Significance is indicated by * p<0.05 vs. mutMMP3 control; # p<005, ## p<0.01 vs. scr-shRNA and actMMP injected rats. (G) Representative photomicrographs of MDA staining in the rat SN sections. Scale bar  = 30 µm. (H) Stereologic counts of MDA-positive neurons in the SN shown as percentage of mutMMP3. ** p<0.01 vs. mutMMP3 injected rats; ## p<0.01 vs. scr-shRNA and actMMP3 injected rats. mutMMP3, mutMMP3/AAV2 particles in the SN; actMMP3, actMMP3/AAV2 particles in the SN; scr-shRNA, injection of scramble shRNA/AAV2 particles into the SN; shNox1, injection of Nox1 shRNA/AAV2 particles into the SN; T17N Rac1, injection of T17N Rac1/AAV2 particles into the SN; +actMMP3, SN injection of actMMP3/AAV2 particles 4 weeks after Nox1 shRNA/AAV2 or T17N Rac1/AAV2.

To investigate toxicity of MMP3 in dopaminergic neurons in the nigrostriatal pathway, we overexpressed actMMP3-tagged with EGFP in the rat SN by stereotaxic delivery of recombinant adeno-associated virus (rAAV2) carrying either actMMP3 or mutMMP3. Similar to MPTP treatment, actMMP3 significantly induced expression of Nox1 in SN DA neurons. Nox1 expressions were determined at 1, 2 and 3 weeks after actMMP3 overexpression. Nox1 expression levels were increased over time by actMMP3 overexpression (Figs. 7A and 7B). To investigate the role of Nox1/Rac1-mediated oxidative stress in actMMP3-induced DA neuronal death, the Nox1/Rac1 system was inhibited by either rAAV2-mediated Nox1 knockdown or dominant negative Rac1 mutant (T17NRac1) expression. Nox1 knockdown efficiency in the SN was verified by Western blot analysis performed at 7 weeks after rAAV2 injection (Fig. 7C and 7D). 4-week pre-injection with either Nox1 shRNA or T17NRac1/AAV viral particles in the SN area significantly diminished SN DA neuronal loss produced by actMMP3 overexpression in the rat SN ( p<0.05) as determined by stereologic counting of tyrosine hydroxylase (TH)-immunostained DA neurons (Figs. 7E and 7F).

Similarly, actMMP3-elicited oxidative stress in DA neurons which was measured by malondialdehyde (MDA) staining were largely attenuated in animals overexpressing either T17N Rac1 or Nox1 shRNA in the SN (Figs. 7G and 7H).

## Discussion

In the present study we show that MMP3 acts upstream of Nox1 activation in dopaminergic neuronal death. MMP3 induced mitochondrial ROS appeared to medicate the Nox1 activation.

MMP3 participates in apoptosis of DA cells caused by toxic stimuli. Cellular MMP-3 activity was elevated under oxidative stress, and this was attenuated by blocking MMP3 [5], [28], [29]. Recent studies have reported the role of MMP3 leading to cell death and neurodegeneration as related to PD. As a protease, the enzyme seems to cleave a number of proteins intracellularly. For example, α-synuclein, the main constituents of Lewy bodies of PD [30] and the protein whose aggregation is known to play a key role in PD pathogenesis [31], is a substrate of MMP3. Upon truncation by MMP3, α-synuclein is left with the highly hydrophobic N-terminal region, which readily forms aggregates and exerts cytotoxicity [32]–[34]. DJ-1, an oxidative stress sensor and a peroxiredoxin-like peroxidase whose gene mutation leads to PD [35], is also cleaved by MMP3, upon which it loses the protective activity [6]. Active MMP3 cleaves Apaf-1 and leads to capase-9 activation [36]. MMP3 contributes to blood brain barrier (BBB) damage and neuroinflammation in PD [37]. We show in the present study that MMP3 induces mitochondrial ROS and leads to Nox1 activation. Therefore, MMP3 seems to cause a number of biochemical changes indside the cell that contributes to cell death.

Dopaminergic neurons are known to be highly susceptible to oxidative stress insults because of their reduced antioxidant capability, high content of dopamine, melanin and lipids, which renders dopaminergic neurons prone to oxidative damage [8]–[10], [38] . ROS have important biological functions and Nox can generate ROS in a regulated manner in several circumstances [39]–[42]. In previous studies, we show that Nox1 serves as a major player in dopaminergic neuronal degeneration in both 6-OHDA- and paraquat-mediated PD rodent models [12], [13]. Nox1 is mainly expressed in colon epithelial cells [43] and it is also reported in various other types of tissues including hepatic stellate cells [44], neuronal cells [13], [45]. Full activation of Nox1 requires the membrane-bound component p22 and the cytosolic partners Nox organizer 1, Nox activator 1, and Rac1 [43].

**Figure 8:**
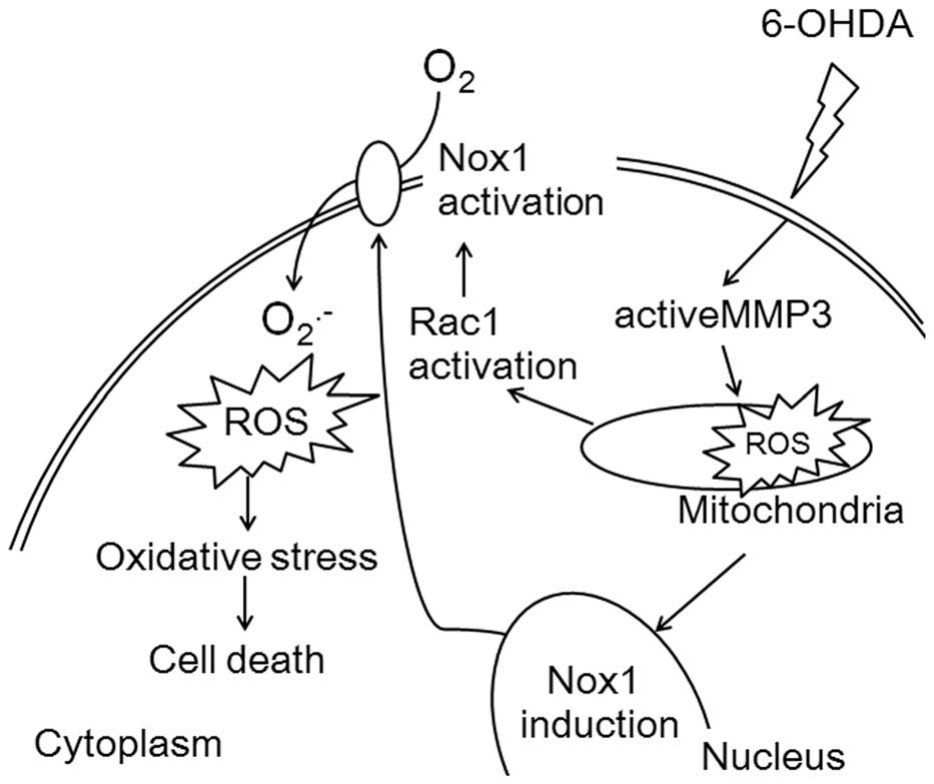
Schematic diagram of the interplay between MMP3 and Nox1 in the process of dopaminergic neuronal death. 6-OHDA induces MMP3 expression and activation. Active MMP3 leads to the mitochondrial ROS production. Nox1 is then induced in a mitochondrial ROS-dependent manner. Subsequently, Nox1 is activated by inducing the Rac1/Nox1 interaction. Consequently, the accumulation of ROS results in increased oxidative stress, and cells lose their viability.

A number of studies have reported that mitochondria are involved in Nox1 expression [14], [46], [47]. In serum deprived 293 cells, mitochondrial ROS at the early phase contributes to Nox1 induction that is responsible for the later phase of ROS accumulation, culminating in cell death [15]. Studies of mitochondrial gene knockout osteosarcoma cells (q0) revealed that the inactivation of mitochondrial genes leads to downregulation of Nox1 and that the transfer of wild-type mitochondrial genes can restore Nox1 expression [14]. Similarly we also showed that mitochondrial respiratory chain inhibitors including rotenone, pyridaben, and antimycin A, increase both mRNA and protein levels of Nox1 [13]. We found that increased level of mitochondrial superoxide in 6-OHDA-treated N27 cells was attenuated by NNGH, MMP3 inhibitor or MMP3 knockdown. Here we identified a novel pathway in which MMP3 activation directly leads to mitochondrial ROS production, causes downstream induction of Nox1, ROS accumulation and consequential DA cell death. Pharmacological inhibition or knockdown of MMP3 attenuated mitochondrial ROS production and Nox1 expression, and overexpression of active MMP3 alone resulted in the mitochondrial ROS production and Nox1 induction. In *in vivo* studies, we demonstrated that *Mmp3* null mice show largely reduced MPTP-induced Nox1 induction and oxidative damage in the SN and rAAV-mediated Nox1 knockdown attenuates actMMP3-mediated dopaminergic neuronal loss. In EMT study, MMP3 induces Rac1b, a constitutively active splicing variant of Rac1 and cellular ROS generation from mitochondria, causing the EMT [17]. Rac1b forms a complex with NADPH oxidase at the membrane and promotes the production of reactive oxygen species, expression of Snail, and activation of the EMT [19]. In accordance with these works, the current work showed that MMP3 plays as an upstream regulator of Nox1 induction via mitochondrial ROS production. Fig. 8 gives a schematic of this process. Therefore, further studies are necessary to elucidate the exact mechanism by which MMP-3 leads to Nox1 activation.

In summary, our study demonstrated that MMP3 activation is a key upstream event that leads to mitochondrial ROS, Nox1 induction, ROS generation, and eventually, results in dopaminergic neuronal death. Therefore, our findings may potentially serve as a novel mechanism for the treatment of PD.
